# *Marsilea crenata* ethanol extract prevents monosodium glutamate adverse effects on the serum levels of reproductive hormones, sperm quality, and testis histology in male rats

**DOI:** 10.14202/vetworld.2021.1529-1536

**Published:** 2021-06-15

**Authors:** Sri Rahayu, Riska Annisa, Ivakhul Anzila, Yuyun Ika Christina, Aries Soewondo, Agung Pramana Warih Marhendra, Muhammad Sasmito Djati

**Affiliations:** Department of Biology, Faculty of Mathematics and Natural Sciences, Brawijaya University, Malang 65145, East Java, Indonesia

**Keywords:** Leydig cell, luteinizing hormone, *Marsilea crenata*, monosodium glutamate, testosterone

## Abstract

**Background and Aim:**

*Marsilea crenata* is an aquatic plant that contains high antioxidants level and could prevent cell damages caused by free radicals. The present study aimed to investigate the effect of *M*. *crenata* ethanol extract on luteinizing hormone (LH), testosterone levels, sperm quality, and testis histology of adult male rats induced by monosodium glutamate (MSG).

**Materials and Methods:**

This study randomly divided 48 male rats into eight groups (n=6): control group; MSG group (4 mg/g body weight [b.w.] for 30 days); MS1, MS2, and MS3 groups (4 mg/g b.w. MSG and *M*. *crenata* ethanol extract at dose 0.216, 0.432, and 0.648 mg/g b.w., respectively, for 30 days); and S1, S2, and S3 groups (*M*. *crenata* ethanol extract at dose of 0.216, 0.432, and 0.648 mg/g b.w., respectively, for 30 days). The blood sample was collected on days 0 and 30 to determine the LH and testosterone levels. The animals were dissected on day 30, and the testes were isolated for morphometric, histology (spermatogenic cell number), and malondialdehyde (MDA) examination. Moreover, semen was collected to determine the sperm quality parameter.

**Results:**

The LH and testosterone levels significantly increased (p<0.05) after *M*. *crenata* administration at all doses. The higher dose of *M*. *crenata* ethanol extract demonstrated a high decrease in MDA level in MSG-treated rat testis; increase of spermatogonia, spermatocytes, spermatids, and Leydig cells number; and increase of seminiferous tubular diameter and germinal epithelium thickness.

**Conclusion:**

The ethanol extract of *M*. *crenata* can improve the levels of LH, testosterone, sperm quality, number of testis morphometric, spermatogenic, and Leydig cells in MSG-treated male rats.

## Introduction

Monosodium glutamate (MSG) is commonly used as a food additive or as a flavor enhancer [1]. MSG is used to improve the taste of salty, sweet, sour, and bitter foods, which is known as umami taste [2]. MSG is classified as a food ingredient that is safe for consumption. However, MSG consumption has increased in Indonesia with an average daily intake of approximately 4.32 g/day [3]. The recommendation of MSG consumption for Asian countries is generally 1.2-1.7 g/day [3,4]. Moreover, excessive use of MSG leads to problems in the male reproductive system caused by reactive oxygen species (ROS) formation in the testis and brain [4]. A previous study demonstrated that intraperitoneal injection of MSG in Wistar rats for 15 and 30 days could reduce testicular weight, change sperm morphology, and decrease testicular ascorbic acid level [5]. Furthermore, MSG administration for 14 days also has an impact on sperm motility, reduction in Leydig cells, interstitial tissue, sperm quantity, and seminiferous tubule change [6]. Another study proved that the high consumption of MSG leads to an increase in Leydig cell necrosis, vacuolization of germ cell, and interstitial tissue loss [7] and also to a decrease in the testosterone hormones in male rats [8]. In addition, MSG is processed into sodium ions and L-glutamate in the body [9]. The L-glutamate is kinesthetically absorbed in the small intestine [2,10,11]. Glutamate catabolism occurs in the cytosol and mitochondria through the transamination process by dehydrogenase enzyme to form a-ketoglutarate when the MSG is transported into the intestinal enterocytes [12]. Free L-glutamate is released into the blood circulation and spreads throughout the body, including the testis and the hypothalamus. The high concentration of L-glutamate activates excessive glutamate receptors, which then increases the intracellular calcium ions (Ca^2+^). An excessive Ca^2+^ amount in the mitochondria would result in ROS production [13,14]. ROS is released in the form of O_2_, which is then converted to hydrogen peroxide (H_2_O_2_). H_2_O_2_ reacts with Fe^2+^ through the Fenton reaction, forming OH^−^, and lipid peroxidation. Lipid peroxidation triggers damages in the cell membranes. Furthermore, lipid peroxidation causes DNA damage in the Leydig cells, which are found in the testis [14]. Leydig cells play an essential role in testosterone secretion in the testis [15]. High ROS levels in the hypothalamus disrupt gonadotropin-releasing hormone (GnRH) production, which then reduces the production of luteinizing hormone (LH) in the anterior pituitary. LH plays a key role in stimulating Leydig cells to produce testosterone [16].

*Marsilea crenata* C. Presl (belongs to the Marsileaceae family), known as water clover, is an aquatic plant that is easily found in Indonesia [17,18]. The leaves of this plant are commonly used as vegetables in Surabaya, Indonesia. The pharmacological activity of the plant includes antineuroinflammation [19], anticholesterol [20], antiosteoporotic [21], and skin aging treatment [22]. The pharmacological studies of *M. crenata* remain limited compared with other species in the Marsileaceae family (e.g., *Marsilea* *minuta* Linn). Furthermore, the research about the effectiveness of *M*. *crenata* on the reproduction system remains unknown. *M*. *crenata* contains a high phytoestrogens compound (e.g., lignans, stilbene, coumestans, coumarin, dihydrochalcone, triterpenoids, and flavones) [22,23]. *M*. *crenata* also contains a high amount of antioxidants, including flavonoids, genistein, and daidzein isoflavones, and Vitamin C [24-26]. The flavonoids contained in *M*. *crenata* prevent membranes from damages caused by free radicals [27]. Vitamin C reduces hydroxyl free radicals and plays a significant role in the biosynthesis of L-carnitine, a compound that contributes to energy production within cells, by accelerating lipid metabolism in the mitochondria [28].

*M*. *crenata* is expected to increase LH and testosterone levels and protects the Leydig cells from damages caused by free radicals due to MSG induction. Rats were used as a model in this study because their genetic, biological, and behavioral characteristics closely resemble those of humans. Moreover, several symptoms of the human condition can be replicated in rats. Hence, this study aimed to investigate the effect of *M*. *crenata* ethanol extract on LH and testosterone levels, sperm quality parameter, and testis histology of adult male rats (*Rattus norvegicus*) with MSG induction.

## Materials and Methods

### Ethical approval

The study was approved by the Animal Care and Use Committee, Brawijaya University, Malang, Indonesia (approval No. 1067-KEP-UB).

### Study period and location

This study was carried out from July 2019 to November 2019 at the Laboratory of Animal Physiology, Department of Biology, Faculty of Mathematics and Natural Sciences, Brawijaya University, Malang, Indonesia.

### Extract preparation

*M*. *crenata* was obtained from the local farmers in Surabaya. The plant was identified by Achmad Mabrur, SKM, M. Kes, Head of UPT Laboratorium Herbal Materia Medica, Batu, Malang, Indonesia and specimen vouchers (074/389/102.7/2017) were deposited in the herbarium of UPT Laboratorium Herbal Materia Medica, Batu, Malang, Indonesia. In addition, 10,000 g of *M*. *crenata* fresh leaves were collected and washed with tap water. The leaves were dried and pulverized into a fine powder. Moreover, 100 g of powdered samples were extracted with 3 L of 70% ethanol at room temperature for 24 h. Samples were filtered and evaporated using a rotary evaporator after 24 h of maceration. The ethanol extract of *M*. *crenata* was stored at 4°C for further analysis.

### Animals

This experimental study used 48 adult male rats (*R*. *norvegicus*) strain Wistar, aged 3-4 months and weighing 200-300 g. The rats were obtained from Malang Murine Farm, Singosari, Indonesia. All animals were kept in standard rat cages (32 cm × 28 cm) at controlled temperature (22±2°C), humidity level 50±5%, and under 12 h light and dark cycle. Each animal was kept in one cage. Animals were fed standard pellet chow and water *ad libitum*. The animals were acclimatized for 2 weeks before and during the study at the Laboratory of Physiology and Animal Structure Development, Department of Biology, Faculty of Mathematics and Natural Sciences, Brawijaya University, Malang, Indonesia.

### Experimental design

This study randomly divided 48 adult male rats into eight groups (n=6). The control group received 15 mL/200 g body weight (b.w.) distilled water orally for 30 days; the MSG group received 4 mg/g b.w. of MSG for 30 days; the MS1, MS2, and MS3 groups orally received 4 mg/g b.w. of MSG for 30 days and *M*. *crenata* extracts at different doses of 0.216, 0.432, and 0.648 mg/g b.w., respectively, for 30 days; then, S1, S2, and S3 groups only orally received *M*. *crenata* ethanol extract at doses of 0.216, 0.432, and 0.648 mg/g b.w., respectively, for 30 days. The *M*. *crenata* dose in rats was calculated on the basis of the conversion of animal doses to human equivalent doses based on body surface area [29]. The MSG dose was referred to in the previous study by Igwebuike *et al*. [30].

The rats were treated with MSG in this study for 15 days before giving with *M. crenata* extract (day 0). The LH and testosterone levels at this time were measured to know the differences between the MSG and control groups. After 15 days of receiving MSG, the rats were continuously treated with MSG and followed by the administration of *M*. *crenata* extract until 30 days (day 30). Both hormones between the MSG and *M*. *crenata* groups were measured at this time.

### Testosterone and LH serum assay

Blood was collected on days 0 and 30. The blood was collected in microtubes and incubated at 37°C for 6 h. The obtained clear sera were stored at −20°C. Testosterone and LH were measured using Rat Testosterone enzyme-linked immunosorbent assay (ELISA) Kit 96T and Rat LH ELISA Kit 96T Bioassay Technology Laboratory, Shanghai, China (catalogue No. E0259Ra) and Rat LH ELISA Kit 96T, Bioassay Technology Laboratory, Shanghai, China (catalogue No.E0179Ra).

### Sperm quality analysis

Animals were sacrificed by neck dislocation and dissected to isolate the epididymis organs at the end of the study (day 30). The cauda epididymis was exposed and incised on one side. Moreover, 1.5 mL of the sperm was quickly sucked and diluted with warm 0.1 M phosphate saline buffer at pH 7.4. Furthermore, 10 µL of sperm sample was assessed for motility, viability, morphology, and sperm concentration. The diluted semen was observed using a microscope with 100× and 400× magnification to observe sperm motility and abnormality. The sperm viability and abnormality were calculated using the following formula:

Viability = (number of live sperm/total of sperm)×100%

Abnormality = (number of abnormal sperm/total of sperm)×100%.

Sperm viability and morphology were determined using eosin nigrosine staining [31]. Thus, 10 μL of diluted semen was added to eosin nigrosine, and a smear was then made. The smear was observed using a microscope with ×400 magnification. To evaluate the sperm concentration, 20 μL of semen was added into 980 μL of fixative solution (1:1 NaHCO_3_ formalin), inserted into hemocytometer, and counted for spermatozoa concentration using a microscope with 400× magnification.

The sperm concentration was calculated using the following formula:

Sperm count = (number of sperm in 5 little square box)×5×10×dilution factor×1000

### Histological examination

Animals were sacrificed through neck dislocation and dissected to isolate the testis at the end of the study (day 30). The histological analysis of the testis was evaluated according to a previously described method [32]. The testis was washed with phosphate-buffered saline and then fixed in 10% formaldehyde for 24 h. The tissue samples were dehydrated in an alcohol solution by follow-up routine methods and embedded in paraffin. Five-micrometer-thick section was stained with hematoxylin–eosin and histologically analyzed using an Olympus BX51 microscope (Olympus Corporation, Inc., New York, NY, USA). The germinal epithelium thickness and the diameter of the seminiferous tubules were investigated with 400× magnification. The assessment of spermatogonia, spermatocyte, spermatid, and Leydig cells was quantified in ten random microscopic fields in the interstitial compartment located between the three tubules.

### Determination of malondialdehyde (MDA) levels in the testis

The MDA levels were measured using the thiobarbituric acid method. The testis was crushed and 0 g of the testis was then homogenated and added with 900 μL of potassium chloride solution, 200 μL of 0.67% trichloroacetic acid, 200 μL of 20% thiobarbituric acid, and 200 μL of hydrogen chloride. The samples were then incubated at 100°C for 15 min and centrifuged at 3000 rpm at 25°C for 10 min. The supernatant was taken to calculate the absorbance value using a spectrophotometer at a wavelength of 535 nm [33].

### Statistical analysis

Statistical analysis was performed using SPSS 16.0 for Windows (SPSS Inc., Armonk, NY, USA). Comparison between groups was analyzed using a one-way analysis of variance (p<0.05) and followed by Tukey’s test. To determine the changes in hormone levels from days 1 to 30, paired sample t-tests were used. Data were represented as mean±standard deviation and p<0.05 was considered statistically significant.

## Results

### LH and testosterone levels

The MSG administration significantly reduced the LH level in male rats on day 0. No significant difference (p>0.05) was noted between LH on days 0 and 30 on the basis of paired sample t-test in most of the groups, except for the MSG and MS3 groups. Meanwhile, the testosterone level showed a similar result with LH compared between days 0 and 30 except for the MSG and MS2 groups (Table-1). Before *M*. *crenata* administration, the LH levels of the *M*. *crenata* groups were not significantly different from the MSG group (day 0; p>0.5). Furthermore, the serum LH levels of the MSG group were significantly decreased on day 30 compared with the control group (p<0.05; Table-1). All groups that received the *M*. *crenata* extract (all doses) shown maintained or increased LH level. Furthermore, the MS3 group (0.648 mg/g b.w.) demonstrated a high LH level.

**Table-1:** Effects of *M. crenata* extract on luteinizing hormone and testosterone serum levels rats exposed to monosodium glutamate and treated with *M. crenata* extract (mean±standard deviation).

Groups	Luteinizing hormone levels (mIU.ml^−1^)		Testosterone levels (nmol.L^−1^)	
	
	0 day	30 days	0 day	30 days
Control	8.89±0.44^b^	8.16±0.36^c^	8.03±0.36^c^	7.40±0.32^b^
MSG	7.54±0.19^a^	6.38±0.40^a*^	6.42±0.40^a^	4.33±0.64^a*^
MS1	7.42±1.64^a^	7.24±0.56^b^	6.13±0.82^a^	7.02±0.37^b^
MS2	7.43±0.73^a^	8.28±0.52^c^	6.87±0.54^a^	7.81±0.51^b*^
MS3	7.65±0.91^a^	8.67±0.62^c*^	6.97±0.70^a^	7.93±0.39^b^
S1	8.23±0.73^b^	8.47±0.58^c^	7.92±0.48^b^	8.27±0.69^c^
S2	8.40±0.67^b^	9.16±0.37^d^	7.75±0.54^b^	8.57±0.77^c^
S3	8.96±1.64^b^	9.97±0.21^d^	8.62±0.72^c^	7.06±0.64^b^

Different letters within the same column show a statistically significant (p<0.05). (*) Were indicated statistically significant between 0 days and 30 days based on paired samples T-test. The control group received 15 μL/200 g b.w. distilled water orally for 30 days; the MSG group received 4 mg/g b.w. of monosodium glutamate for 30 days; the MS1, MS2, and MS3 orally received with 4 mg/g b.w. of monosodium glutamate for 30 days and also orally received *M. crenata* extracts at different doses of 0.216 mg/g b.w., 0.432 mg/g b.w., and 0.648 mg/g b.w., respectively for 30 days; S1, S2, and S3 only orally received *M. crenata* extracts at doses of 0.216 mg/g b.w., 0.432 mg/g b.w., and 0.648 mg/g b.w., respectively, for 30 days. *M. crenata=Marsilea crenata*

The results also showed that the testosterone levels in the MSG group were significantly decreased on day 30 compared with the control group (p<0.05; Table-1). All doses of *M*. *crenata* extracts demonstrated a significant increase in testosterone levels on day 30. The highest testosterone levels were found in the S2 (0.432 mg/g b.w.) and S1 (0.216 mg/g b.w.) groups.

### Testis histology

The administration of *M*. *crenata* extract in MSG-treated rats exhibited the highest number of all parameters of the morphometric seminiferous tubules compared with other MSG groups. The MSG group led to a decrease in tubular diameter in rat testis compared with that in the control group (Table-2). The administration of *M*. *crenata* extract in MSG-treated rats led to an increase in tubular diameter in a dose-dependent manner. The mean tubular diameter increased in all *M*. *crenata* groups compared with that in the control group. Furthermore, the germinal epithelium thickness of the seminiferous tubules in MSG-treated rats exhibited a decrease compared with that in the control group. In all groups of animals receiving *M*. *crenata* extract, the germinal epithelium thickness was significantly higher than in the MSG group (p<0.05). In rats administered only with *M*. *crenata*, the diameter of the seminiferous tubules and germinal epithelium thickness was not significantly different compared with that in the control group (p>0.05). The administration of *M*. *crenata* extract in normal rats did not alter the morphometric of the seminiferous tubules.

**Table-2:** Effect of *M. crenata* extract on morphometric parameters of rats exposed to monosodium glutamate and treated with *M. crenata* extract.

Groups	Morphometric of seminiferous tubules (µm) (mean±standard deviation)	

	Seminiferous tubules diameter	Germinal epithelium thickness
Control	250.09±2.57^b^	50.33±0.88^b^
MSG	214.69±2.04^a^	45.81±0.05^a^
MSG+S1	264.94±3.65^c^	50.96±1.28^b^
MSG+S2	269.34±4.17^c^	51.40±0.53^b^
MSG+S3	270.01±5.97^c^	52.84±0.53^b^
S1	251.05±3.75^b^	51.61±1.05^b^
S2	254.01±5.59^b^	53.89±0.43^b^
S3	260.02±3.43^b^	54.19±0.51^b^

Different letters within the same column show a statistically significant (p<0.05). *M. crenata=Marsilea crenata*

The mean of the spermatogonia, spermatocytes, and spermatids was significantly reduced in the MSG-treated group compared with that in the control group (p<0.5; Table-3). Moreover, the number of Leydig cells significantly reduced in the MSG-treated group compared with that in the control group (p<0.05). The administration of *M*. *crenata* extracts in MSG-treated rats significantly increased the number of spermatogonia, spermatocytes, spermatids, and Leydig cells in a dose-dependent manner. The administration of *M*. *crenata* extract in the S1 and S3 groups significantly increased the number of Leydig cells. However, the number of Leydig cells in the S3 group was similar to that in the control group.

**Table-3:** Effect of *M. crenata* extract on the number of spermatogenic cells in rats exposed to monosodium glutamate and treated with *M. crenata* extract.

Groups	Spermatogenic cells (mean±standard deviation)			

	Spermatogonia	Spermatocyte	Spermatid	Leydig
Control	57.33±5.12^b^	63.83±5.41^b^	162.50±6.38^c^	8.55±0.37^b^
MSG	46.08±2.81^a^	54.00±2.75^a^	113.00±11.80^a^	4.03±0.43^a^
MS1	57.25±4.99^b^	63.58±4.65^b^	126.75±12.26^b^	7.13±0.32^b^
MS2	57.75±2.67^b^	65.42±1.85^b^	131.50±11.72^b^	7.68±0.64^b^
MS3	58.92±0.96^b^	69.17±4.39^b^	162.17±8.12^c^	8.23±0.38^b^
S1	60.50±3.45^b^	64.83±2.08^b^	163.25±4.25^c^	9.18±0.24^c^
S2	61.75±3.41^b^	68.58±3.00^b^	166.00±3.74^c^	10.58±0.17^c^
S3	67.17±2.65^c^	79.17±1.77^c^	189.92±8.97^d^	7.08±0.34^b^

Different letters within the same column show a statistically significant (p<0.05). *M. crenata=Marsilea* crenata

**Figure-1:**
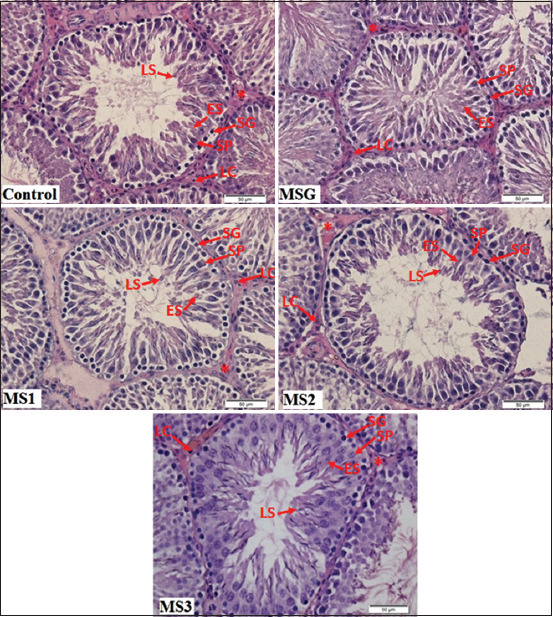
Seminiferous tubule section in rats. (H & E staining [400× magnification]). H & E staining showing the seminiferous tubules and the interstitial tissue (asterisks) containing the Leydig cells (LC). Each tubule is lined with Sertoli cells (SC), spermatogonia (SG), primary spermatocytes (SP), early spermatids (ES), and late spermatids (LS). In the MSG rats, tubular lumen filled with detached germ cells. The administration of *Marsilea crenata* showed improvement of spermatogenesis.

**Figure-2:**
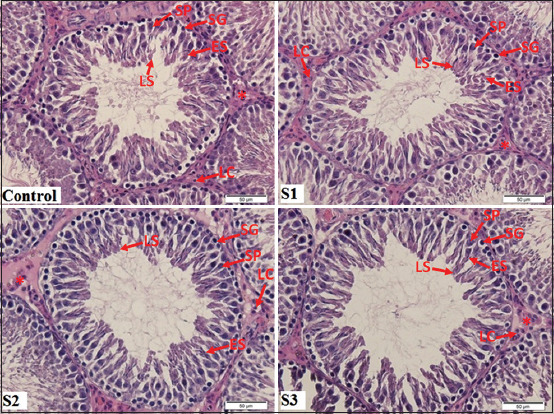
Seminiferous tubule section in rats. (H & E staining [400× magnification]). H & E staining showing the seminiferous tubules and the interstitial tissue (asterisks) containing the Leydig cells (LC). Each tubule is lined with Sertoli cells (SC), spermatogonia (SG), primary spermatocytes (SP), early spermatids (ES), and late spermatids (LS). The seminiferous tubules of control group showed germ cells organized in concentric layers and tubular lumen was empty. The administration of only *Marsilea crenata* in healthy rat was not significantly different with control group in the spermatogenesis condition.

The seminiferous tubules in the testicular hematoxylin–eosin section of the control group with normal shape showed germ cells organized in concentric layers, and the tubular lumen was empty (Figure-1). Moreover, seminiferous tubules in the testicular section of MSG-treated rats showed disarranged epithelial layers and tubular lumen filled with detached germ cells. The morphology of the Leydig cells was a regular shape with a polyhedral shape in both the control and *M*. *crenata* groups. Normal Leydig cells were arranged in clusters in the interstitial space between the seminiferous tubules. The *M*. *crenata* administration in MSG-treated rat showed altered seminiferous tubules, which were in regular shape, and the tubular lumen was empty. The use of only *M*. *crenata* in the healthy rat was not significantly different from that in the control group (Figure-2), which indicated that *M*. *crenata* did not exhibit an alteration in the spermatogenesis process of healthy rat testis.

### Sperm quality

The sperm viability in the control group was approximately 61.08±4.44% (Table-4). A highly significant reduction in the sperm viability of approximately 39.86±10.39% was confirmed after MSG induction for 30 days. Interestingly, all doses of *M*. *crenata* extract in MSG-treated rats exhibited a significant increase in sperm viability. A highly significant rise in sperm viability of approximately 74.99±3.17% was observed in MSG-treated rats, which received a high dose of *M*. *crenata* MS3.

**Table-4:** Effect of *M. crenata* extract on the quality of semen in rats exposed to monosodium glutamate and treated with *M. crenata* extract.

Groups	Semen quality (mean±standard deviation)			

	Motility (%)	Viability (%)	Abnormalities (%)	Semen concentration (10^6^/ml)
Control	76.75±9.25^c^	61.08±4.44 ^b^	9.86±1.63 ^a^	62.50±3.68 ^b^
MSG	63.75±7.50^b^	39.86±10.39^a^	37.83±2.22^b^	48.13±4.49^a^
MS1	48.75±6.88^a^	62.05±3.84^b^	10.46±1.10^a^	61.88±4.83^b^
MS2	55.00±2.04^a^	68.05±3.15^b^	8.63±0.39^a^	76.88±8.50^c^
MS3	75.00±4.08^c^	74.99±3.17^b^	4.80±0.30^a^	80.00±3.23^c^
S1	71.25±5.54^c^	73.19±1.90^b^	10.09±1.23^a^	63.75±1.61^b^
S2	73.75±4.73^c^	76.15±0.68^b^	6.32±0.69^a^	66.25±2.98^b^
S3	60.00±4.56^b^	71.07±2.91^b^	5.99±0.35^a^	81.88±4.94^c^

Different letter within the same column showed a statistically significant (p<0.05). *M. crenata=Marsilea crenata*

The sperm motility percentage was significantly decreased after MSG induction compared with the control group (Table-4). A high dose of *M*. *crenata* extract (MS3) significantly increased sperm motility after MSG induction. Moreover, sperm abnormalities were mostly found in MSG-treated rats compared with healthy rats. All doses of *M*. *crenata* extract exhibited a significant decrease in sperm abnormality. A high reduction of sperm abnormalities was found in the MS3 group. This study also found that the sperm number was decreased after MSG induction. *M*. *crenata* extract could ameliorate the sperm number at all doses. Moreover, a high dose of *M*. *crenata* extract (MS3) showed a high increase in the sperm number.

### MDA levels in testis

**Figure-3:**
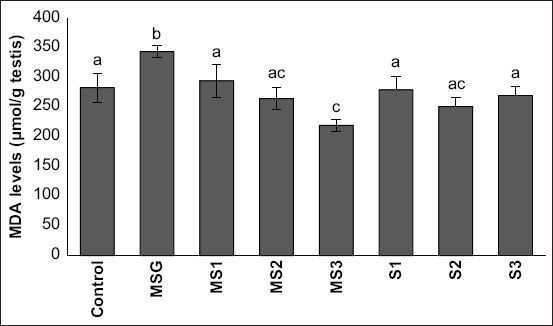
The effect of *Marsilea* *crenata* on the malondialdehyde level in the testis of monosodium glutamate-treated rat. Data are presented as mean±standard deviation. The different letters show a significant change. The control group received 15 μL/200 g body weight (b.w.) distilled water orally for 30 days; the MSG group received 4 mg/g b.w. of monosodium glutamate for 30 days; the MS1, MS2, and MS3 orally received 4 mg/g b.w. of monosodium glutamate for 30 days and also orally received *M. crenata* extracts at different doses of 0.216 mg/g b.w., 0.432 mg/g b.w., and 0.648 mg/g b.w., respectively, for 30 days; S1, S2, and S3 only orally received *M. crenata* extracts at doses of 0.216 mg/g b.w., 0.432 mg/g b.w., and 0.648 mg/g b.w., respectively, for 30 days.

The MSG group had high levels of MDA in male rat testis compared with the control group ([344.25±10.28] mmol/mL vs. [283.63±24.18] mmol/mL). The administration of *M*. *crenata* extracts at a dose of 0.648 mg/g b.w. significantly reduced MDA levels in MSG-treated rats (124.37 mmol/mL) compared with other *M*. *crenata* groups. The higher dose of *M*. *crenata* extract demonstrated a high decrease in MDA levels in the testes of MSG-treated rats (Figure-3). The administration of only *M*. *crenata* at all doses did not alter the MDA level in rat testis.

## Discussion

LH is the primary hormone for Leydig cell stimulation to produce testosterone [34]. The LH concentration in the MSG group on day 45 was found to be significantly lower than in the control group. The results are in line with the research by Park *et al*. [35] who found that MSG administration at a dose of 4 mg/g b.w. in adult mice leads to a decline in the number of neurons and a decrease in GnRH production. This condition causes a decrease in LH production in the anterior pituitary. Furthermore, Ochiogu *et al*. [36] confirmed that oral and subcutaneous MSG administration at a low dose (0.25, 0.50, and 1.00 mg/g b.w.) for 14, 28, and 42 days in male rats significantly reduced the levels of GnRH, LH, testosterone, and total cholesterol, respectively.

The testosterone level in the MSG group was lower than in the control group because of the decrease in Leydig cell number. This decline may indirectly influence LH concentration. Increased ROS in the testicles due to MSG induction, which triggers lipid peroxidation, damages the cell membranes, especially phospholipid content, and causes ruptures and necrosis in the membranes [14,34]. A decreased number of Leydig cells is followed by a decrease in testosterone production and testosterone levels in the testicles [37]. According to Igwebuike *et al*. [30], oral MSG administration at a dose of 4 mg/g b.w. in Sprague–Dawley rats every 48 h for 6 weeks significantly reduced the testosterone serum levels.

Testosterone concentrations in the *M*. *crenata* extract groups on day 30 were significantly increased compared with those in the MSG group. It may be caused by Vitamin C content in *M*. *crenata* extract that inhibited free radicals from MSG. Vitamin C reduces hydroxyl free radicals and plays a significant role in L*-*carnitine biosynthesis. This compound contributes to the production of energy inside the cells by accelerating lipid metabolism in mitochondria [28]. Vitamin C binds to hydroxyl radicals by releasing its electrons from two bonds between the second and third carbon molecules out of six-carbon units. After releasing the particles to free radicals, Vitamin C will be oxidized to ascorbic radicals that are relatively more stable than free radicals [38]. Furthermore, isoflavones in *M*. *crenata* can inhibit the 5a-reductase that catalyzes testosterone into 5a-dihydrotestosterone, and CYP19 (aromatase) that also mediates the conversion of testosterone into estradiol. Conversely, aromatase activity becomes intensive enough to increase estradiol conversion when isoflavone concentrations are high [28,39].

This study showed that a high dose of *M*. *crenata* exhibited a high decrease in MDA levels in MSG-treated rats. *M*. *crenata* extract administration for 30 days was able to suppress MDA formation in rat testis due to excessive MSG consumption. Some types of flavonoids contained in *M*. *crenata*, namely, quercetin, genistein, and daidzein, can act as antioxidants [40]. Moreover, quercetin can bind to other molecules as reduction agents for free atoms, hydrogen donators, singlet oxygen quenchers, superoxide radical scavengers, and metal chelators because quercetin is a flavonol derivative from members of flavonoids that have phenolic hydroxyl groups [41]. Genistein reacts with peroxyl radicals to inhibit lipid peroxidation [42]. A coumarin is a phenolic group composed of benzene and a-pyrone rings. Furthermore, coumarin has the potential as nitric oxide scavenging, which directly reacts with nitric oxide so that it can suppress nitrite formation [43]. β-carotene can act as antioxidants as peroxyl and singlet oxygen radical scavenger, which deactivates peroxyl radicals and oxygen singlets by reacting with them to form stable carbon resonances to prevent cellular lipid damage [44].

Leydig cells play crucial roles in the secretion of testosterone hormones, whose activities were regulated by LH [15]. The number of Leydig cells in the MSG group was found fewer than the control group. It may be caused by an increased level of free radicals in the testis. High levels of L-glutamate trigger the overactivation of glutamate receptors and lead to irregularities in the mitochondrial electron chain function [8]. This condition disrupts the membrane permeability and adenosine triphosphate synthesis, which eventually damages the Leydig cells [45,46]. The decrease in the number of Leydig cells also affects the production of testosterone hormones in the testis. Sarhan [37] showed that the MSG administration at a dose of 6 mg/g b.w./day for 45 days in male rats increased ROS production, which is demonstrated by a significant increase in the MDA level. Furthermore, Leydig and spermatogenic cells showed irregular nuclei, cytoplasmic vacuolation, and swelling in the mitochondria [47].

The *M*. *crenata* group at dose 0.648 mg/g b.w. exhibited a high number of Leydig cells compared with the MSG group. It was caused by *M*. *crenata* contents, including Vitamin C, which inhibited the oxidants (free radicals) from MSG and protected the Leydig cells from getting damaged [28]. It also may be caused by genistein and daidzein isoflavones, which have similar features to estrogen or are known as phytoestrogens [37,48]. Phytoestrogens enhance the cell proliferation and maturation process by activating the estrogen-responsive element gene, resulting in a higher number of cells in the testicular organ [26]. Flavonoids in *M*. *crenata* also prevent membrane cell and DNA from damages due to free radicals [46].

## Conclusion

The use of *M*. *crenata* extract can improve the levels of LH, testosterone, sperm quality, and number of testis morphometric, spermatogenic, and Leydig cells in rats treated with MSG. It seems that a high antioxidant level and the active compound contained in *M*. *crenata* can improve the negative effect of MSG. Some possible limitations in this study may exist. The study suggests that more methodological work is promising for future research to estimate cellular ROS levels and assess the antioxidant status in the testes.

## Authors’ Contributions

SR: Designed the study and drafted the manuscript. RA and IA: Conducted the study and assisted the sample collection. YIC: Helped the interpretation of the results. AS and APWM: Supervised the histology analyses. MSD: Revised the manuscript. All authors read and approved the final manuscript.
